# The effectiveness of the What Do You Drink (WDYD) web-based brief alcohol intervention over time: a two-arm parallel group randomized controlled trial applying an ecological momentary assessment approach

**DOI:** 10.1186/1940-0640-8-S1-A82

**Published:** 2013-09-04

**Authors:** Carmen Voogt, Emmanuel Kuntsche, Marloes Kleinjan, Evelien Poelen, Lex Lemmers, Rutger Engels

**Affiliations:** 1 Behavioural Science Institute, Radboud University, Nijmegen, the Netherlands; 2 Addiction Switzerland, Research Institute, Lausanne, Switzerland; 3 Trimbos Institute, Netherlands Institute of Mental Health and Addiction, Utrecht, the Netherlands

## 

The aim of the current study was to test whether the web-based brief alcohol intervention entitled “What Do You Drink” (WDYD) can sustain a reduction in alcohol use among heavy drinking students after one, three, and six months follow-up. A two-arm parallel group randomized controlled trial applying an ecological momentary assessment (EMA) approach with 30 data time-points was conducted in the Netherlands, 2010-2011. Students aged 18 to 24 years old who reported heavy drinking in the past six months and who were ready to change behaviour were included in the study. Participants were randomized into the experimental (*n* = 456: WDYD intervention) or control condition (*n* = 451: no intervention). Outcome measures were weekly alcohol consumption and frequency of binge drinking. Latent growth curve analyses to model individual change in alcohol use over time by condition revealed that participants in the experimental condition had significantly lower weekly alcohol consumption and frequency of binge drinking compared to participants in the control condition that sustained after three (B_0_ = -2.60, *p* < .001; B_1_ = 0.16, *p* = 0.08) and six (B_0_ = -0.14, *p* = 0.01; B_1_ = 0.00, *p* = 0.19) months follow-up, respectively. Additional linear regression analyses indicated that the intercept differences resulted from an increase in alcohol use for participants in the control condition compared to those in the experimental condition. The WDYD intervention was shown to be effective in preventing an increase in weekly alcohol consumption and frequency of binge drinking shortly after the intervention that sustained after three and six months post-intervention, respectively. Trial registration: Netherlands Trial Register NTR2665.

